# CropSight: a scalable and open-source information management system for distributed plant phenotyping and IoT-based crop management

**DOI:** 10.1093/gigascience/giz009

**Published:** 2019-01-31

**Authors:** Daniel Reynolds, Joshua Ball, Alan Bauer, Robert Davey, Simon Griffiths, Ji Zhou

**Affiliations:** 1 Engineering Biology, Earlham Institute, Norwich Research Park, Colney Lane, Norwich, NR4 7UZ, UK; 2 Plant Phenomics Research Center, China-UK Plant Phenomics Research Centre, Nanjing Agricultural University, No 1, Weigang, Nanjing, Jiangsu Province, China, 210095; 3 Crop Genetics, John Innes Centre, Norwich Research Park, Colney Lane, Norwich, NR4 7UH, UK; 4 University of East Anglia, Norwich Research Park, Norwich, NT4 7TJ, UK

**Keywords:** CropSight, distributed plant phenotyping, phenomics, IoT-based crop management, information system

## Abstract

**Background:**

High-quality plant phenotyping and climate data lay the foundation for phenotypic analysis and genotype-environment interaction, providing important evidence not only for plant scientists to understand the dynamics between crop performance, genotypes, and environmental factors but also for agronomists and farmers to closely monitor crops in fluctuating agricultural conditions. With the rise of Internet of Things technologies (IoT) in recent years, many IoT-based remote sensing devices have been applied to plant phenotyping and crop monitoring, which are generating terabytes of biological datasets every day. However, it is still technically challenging to calibrate, annotate, and aggregate the big data effectively, especially when they were produced in multiple locations and at different scales.

**Findings:**

CropSight is a PHP Hypertext Pre-processor and structured query language-based server platform that provides automated data collation, storage, and information management through distributed IoT sensors and phenotyping workstations. It provides a two-component solution to monitor biological experiments through networked sensing devices, with interfaces specifically designed for distributed plant phenotyping and centralized data management. Data transfer and annotation are accomplished automatically through an hypertext transfer protocol-accessible RESTful API installed on both device side and server side of the CropSight system, which synchronize daily representative crop growth images for visual-based crop assessment and hourly microclimate readings for GxE studies. CropSight also supports the comparison of historical and ongoing crop performance while different experiments are being conducted.

**Conclusions:**

As a scalable and open-source information management system, CropSight can be used to maintain and collate important crop performance and microclimate datasets captured by IoT sensors and distributed phenotyping installations. It provides near real-time environmental and crop growth monitoring in addition to historical and current experiment comparison through an integrated cloud-ready server system. Accessible both locally in the field through smart devices and remotely in an office using a personal computer, CropSight has been applied to field experiments of bread wheat prebreeding since 2016 and speed breeding since 2017. We believe that the CropSight system could have a significant impact on scalable plant phenotyping and IoT-style crop management to enable smart agricultural practices in the near future.

## Background

Automated phenotyping technology has the potential to enable continuous and precise measurement of dynamic phenotypes that are key to today's plant research [1, 2]. Quantitative phenotypic traits collected through crop development are not only important evidence for plant scientists to understand the dynamics between plant performance, genotypes, and environmental factors (i.e., genotype-environment interaction [GxE]), they are critical for agronomists and farmers to closely monitor crops in fluctuating agricultural conditions [3–5]. High-quality phenotyping and climate datasets lay the foundation for meaningful phenotypic analysis, which is likely to produce an accurate delineation of the genotype-to-phenotype pathway for the assessment of yield potential and environmental adaptation [6, 7]. Presently, although many automated phenotyping platforms are capable of generating large plant-environment data [8], it is still technically challenging to collect, calibrate, annotate, and aggregate these datasets effectively, especially for experiments carried out in multiple locations and at different scales [9, 10].

lWith the rise of Internet of Things (IoT) technologies and their applications in plant phenotyping [11], a number of commercial data and experiment management solutions have been developed on the basis of customized hardware and proprietary software. For example, LemnaTec's Field Scanalyzer platform [12] employs a simple hypertext transfer protocol (HTTP) server with an SQLite database to facilitate crop monitoring and deep phenotyping using LemnaControl and LemnaBase systems [13, 14]. Integrated Analysis Platform (LemnaTec) [15] together with LemnaGrid analysis software form an automated data processing platform that combines raw image collection, metadata association, and phenotypic analysis for indoor plant phenotyping. Phenospex's FieldScan system uses an infield WiFi network to connect PlantEye three-dimensional laser scanners, climate sensors, and a gantry system with a PostgreSQL database to realize the scanner-to-plant phenotyping [16]. Furthermore, the PlantScreen system (Photon Systems Instruments) manages fluorescence images through computer vision techniques via dedicated networks and databases [17]. However, the above-mentioned commercial systems require ongoing licensing maintenance and additional costs for developing new functions. It is therefore challenging for a broader plant research community to adopt and extend them easily in order to meet the growing needs of today's plant research [10].

Recently, some research-based systems have also been introduced to the scientific community. For example, by combining local and global management subsystems, a cloud-based remote control system has been developed to monitor environmental conditions in tropical horticulture cultivation as well as remotely control drip irrigation for tomato plants based on soil moisture content [18]. The framework has been tested under unstable network connections in rural areas, which has demonstrated its potential and usefulness. However, it requires long-term outdoor verification and still has compatibility issues when integrating with different sensing devices. PhotosynQ software manages data collection and storage through a handheld device called MultispeQ [19]. It uses Bluetooth to retrieve leaf surface images, environmental and geolocational data collected by the handheld device, which are then stored in a mobile phone or a laptop for centralized analysis. The system requires manual interference for data synchronization and onsite workstations or cloud-based servers for data analysis. Hence, it is tailored for small-scale and qualitative phenotyping tasks. BreedVision is another system that gathers data through a network-based HTTP server [20]. Mounting multiple sensors on a tractor, BreedVision is used to carry out field phenotyping for wheat breeding. Sensors communicate to a structured query language (SQL) database running in an embedded system. However, similar to the above-mentioned commercial systems, this platform is designed for bespoke hardware and has not provided an open application programming interface (API) that allows external hardware and software to connect. Solely for collecting climate datasets, the PANGEA architecture [21] was successfully established to network large numbers of connections (e.g., wireless sensor networks [WSNs]) for agricultural practices [22]. This system has been used to integrate large-scale WSN installations through open and distributed smart device interfaces. However, it cannot handle image-based datasets and thus limits its applications in image-based plant research. Recently, a comprehensive and open-source phenotyping hybrid information system (PHIS) has been developed by INRA [23]. The PHIS aims to provide a platform to enable data tracing and reanalysis of phenomic data (for both sensor- and image-based data) collected on thousands of plants, sensors, and events. It can identify and retrieve objects, traits, and relations via ontologies and semantics. Because the PHIS needs to incorporate many external phenotyping and modeling systems, it is heavyweight and suitable for post-experimental data integration and analysis.

The above-mentioned industrial and academic efforts identify the need to develop a scalable and openly available information management system to deal with our growing experimental needs and biological datasets. It needs to handle different types of datasets acquired in plant phenotyping experiments. To integrate data transfer, calibration, annotation, and aggregation effectively, such a system should be flexible for changeable experimental designs and expandable with third-party hardware and external software. More importantly, the system needs to enable users to closely monitor experiments conducted in different locations while experiments are being carried out.

With these design requirements in mind, we developed CropSight, a scalable IoT-based information management system that is easy to use and flexible to deploy in diverse experimental scenarios. CropSight is an open-source software system that provides a range of interfacing options for the community to adopt and extend. We followed a distributed systems design during the development so that experimental, phenotypic, and environmental data collected from infield and indoor experiments could be integrated efficiently. The system provides a unified web interface for users to oversee data collection, calibration, and storage on a regular basis. Through our three-year wheat prebreeding field experiments (2016–2018) [24] and the speed breeding project [25], a powerful visualization component and a flexible data/experiment management solution has been established. Equipped with CropSight, users can now closely monitor different experiments, both ongoing and historic, running in different locations. Furthermore, the modulated software architecture has made it possible to change scale and performance for growing experimental needs. To our knowledge, the research-based CropSight system has the potential to significantly contribute toward dynamic data collation and scalable experimental management for both plant phenotyping and crop GxE studies.

## Findings

IoT is a fast-growing field. IoT-based sensors are generating terabytes of data for plant research and agriculture services every day [26]. Since the existing data/experiment management solutions heavily rely on bespoke data collection approaches, they cannot be easily adopted and extended. Also, most of the present solutions require the construction of a centralized management system, which could not resolve the problem of scalability and accessibility because the distributed nature of IoT technologies and the centralized data administration infrastructure are likely to confound each other. Instead, we developed a two-component solution. The first part of this is a device-side system that is lightweight and capable of interacting directly with distributed IoT sensing devices, which can ensure onboard data standardization and data collection. The second component is a server-side system that collates and stores image- and sensor-based data, with SQL as the back-end. This server-side system is more comprehensive and responsible for managing and visualizing dynamic crop-environment data collected during experiments. Combining both parts, the open-source CropSight system is capable of bringing scalability and flexibility to users.

### The systems design

**Figure 1:**
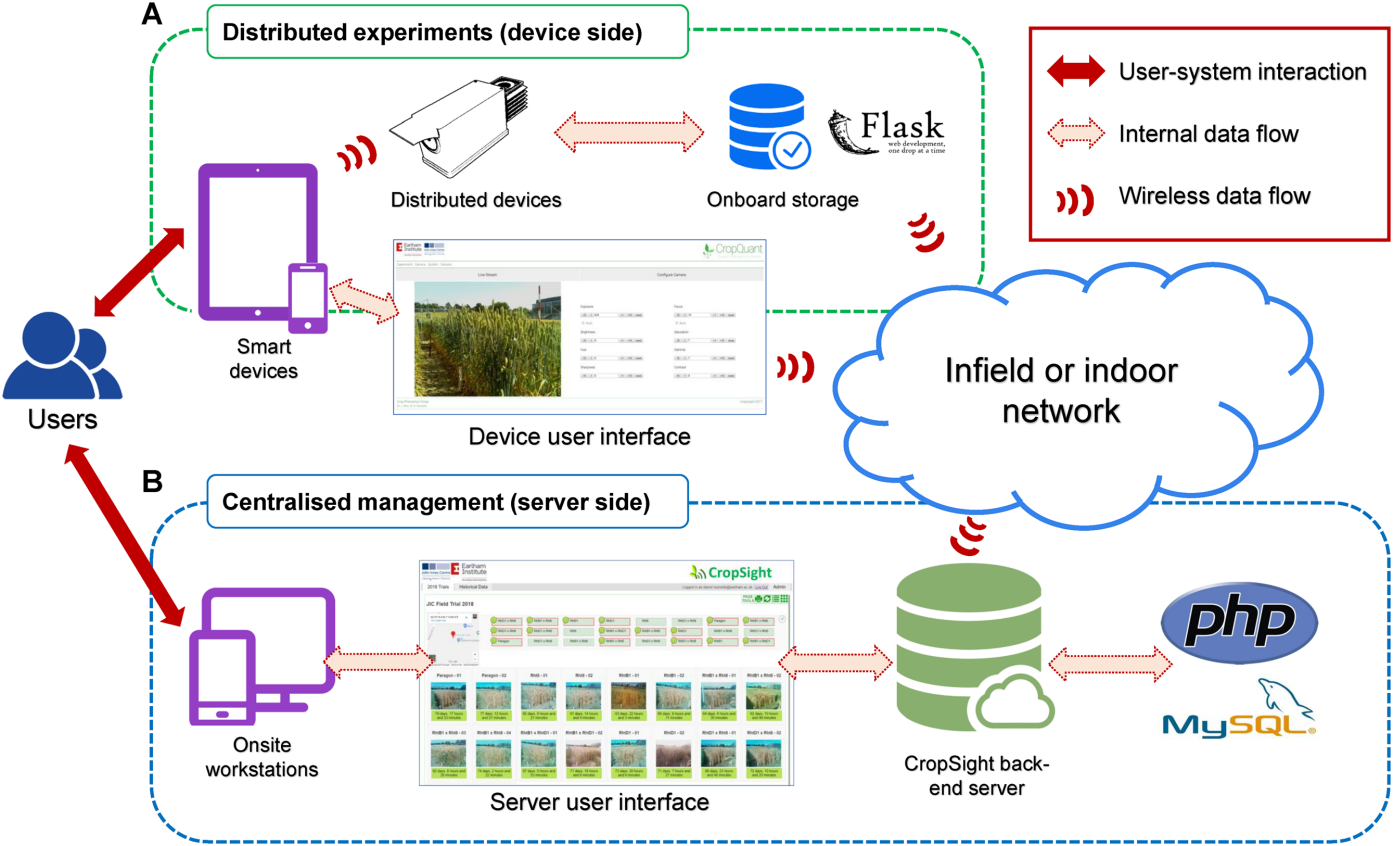
A deployment diagram of the CropSight system in biological experiments. **(A)** CropSight facilitates users to interact with distributed infield or indoor phenotyping devices using wired (i.e., ethernet cables) or wireless connection (e.g., WiFi network). The CropSight client running on distributed workstations supports remote control and onboard data management. **(B)** Users can connect, monitor, and administer experiments through the centralized CropSight server in near real time. Through dedicated networks, the CropSight back-end server collates and integrates large-scale image- and sensor-based phenotyping datasets in an SQL database.

The two-component systems design of CropSight is shown in Fig. 1. We used a Python-based web framework, Flask [27, 28], as the base for the device-side services. The main reason for this choice is that Python, a high-level programming language widely used by the scientific community, can interact with many single-board computers (e.g., a *Raspberry Pi* computer) commonly embedded in distributed IoT sensors and/or phenotyping devices. This framework administers onboard data flow and storage together with a lightweight server for web-based interactions (Fig. 1A). As Flask is hardware independent, the approach can be applied to any hardware that supports Python. Additional services such as Linux *crontab* scheduling system, dynamic host configuration protocol (used for establishing self-operating WiFi network), and virtual network computing (VNC) services can also be easily added or removed to maintain the simplicity of the device-side system.

Powered by Hypertext Pre-processor (PHP) 5+ [29] and MySQL [30], the device-side system can facilitate real-time interactions between smart devices (e.g., smartphones and tablets) and IoT devices. The graphic user interface (GUI) was developed using PHP and JavaScript, which can be opened in a web browser such as Chrome and Firefox on any smart device. A PHP-based RESTful API [31] was adopted to regulate hourly client-server communications. A lightweight SQL server, MariaDB [32], was used for collecting and storing different formats of datasets, including images, climate sensors, and experimental settings. The device-side system can give access to each phenotyping device so that live video streaming and remote system configuration can be initiated by users to deploy phenotyping devices (Supplementary Fig. S1) as well as to establish indoor or infield experiments just using a smartphone or a tablet. Also, the GUI allows users to enter metadata including trials, experiments (e.g., genotypes, treatments and biological replicates), and brief descriptions, while phenotyping devices are being installed. The distributed IoT-based design has massively improved the mobility and flexibility of phenotyping tasks.

The server-side system bridges the connection between data aggregation and cloud-based interfacing (Fig. 1B). This approach facilitates biological data acquired at different locations to be synchronized with a centralized server for data management, detailed traits analyses, and decision making in crop management. PHP5+ was used to develop the system that supports Apache and an SQL server such as MySQL [30]. The server-side system initiates regular updates of the status of each distributed IoT device via server user interface, with information such as online or offline status of the device, operational mode, representative daily images, micro-climate readings, and the usage of computing resources (i.e., central processing unit and memory). Between 2016 and 2018, the two-component CropSight system has been successfully applied to monitor wheat prebreeding experiments in the field and indoor wheat speed breeding (i.e., growth chamber and greenhouse) simultaneously (Supplementary Fig. S2).

**Figure 2:**
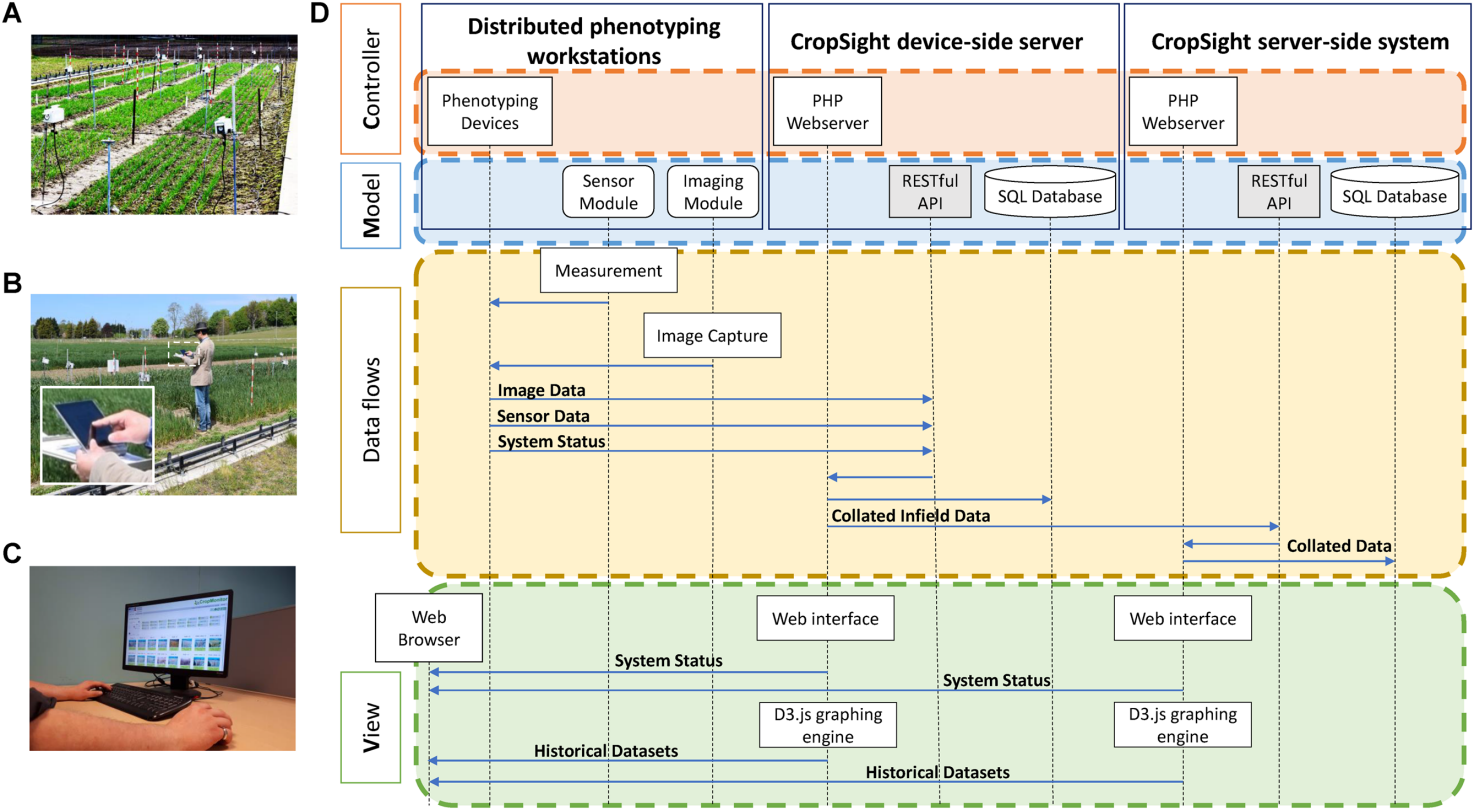
A component diagram of the deployment, detailed data flows, and device- and server-side applications of the CropSight system. **(A)** IoT phenotyping workstations installed in wheat field experiments. Distributed phenotyping nodes are connected by the CropSight system. **(B)** Infield phenotyping devices can be directly accessed and controlled through the device-side CropSight system using a smart device. **(C)** The server-side CropSight system can be used to manage ongoing indoor and infield experiments by accessing a centralized web interface. **(D)** A detailed component diagram showing the MVC design of CropSight and the interface between distributed phenotyping workstations, device-side CropSight server, server-side system, and detailed data flows. The data input is through a RESTful API, responsible for transferring data between servers and enabling interactions through a web-based user interface.

While CropSight is designed to allow use by individuals with no technical background, the installation of the system still requires an IT technician to complete (see Additional File 1 for detailed instructions). To install the system, a functioning PHP and SQL server is required. Also, as it needs to run on a network-enabled web server, a network infrastructure is therefore required to operate CropSight (Fig. 2). Due to the rural location of many crop research experiments, it is often expensive and unfeasible to install wired or wireless networks in some experimental sites. Hence, our solution is to establish an *ad hoc* and self-operating network through universal serial bus (USB) WiFi dongles mounted on IoT devices, e.g., a CropQuant phenotyping workstation [24], so CropSight can manage data transfer between distributed devices (distributed nodes) and a central server (a server node). The self-operating network can be either a Star or a Mesh network topology (Supplementary Fig. S3). In our case, we have established a Star network typology in field experiments of bread wheat. The device-side CropSight system administers the self-operating network, enabling peer-to-peer HTTP accessing points to network distributed nodes for data calibration and synchronization (Fig. 2A) or to establish a direct link between a smart device and a server node (Fig. 2B). After correlating and collecting all data from the device side, the system will then transfer the data to the server-side system, where users could oversee different experiments at near real time (Fig. 2C). The self-operating networking approach enables flexible WiFi coverage over experiment sites. It is important to point out that the effective radius of one Star network in our experiments is around 1,000–1,200 m^2^, which is determined by the effective 25-meter range of the USB WiFi dongles installed in our CropQuant phenotyping workstations. A normal Star network includes eight low-cost distributed nodes and one server node, which costs approximate $4,200 to build in-house. For an individual phenotyping workstation (i.e., a distributed node), around 20 GB sensor- and image-based data could be generated in a growing season.

### An MVC architecture

When implementing the CropSight system, we followed Model-view-controller (MVC) software architecture, dividing the system into three interconnected parts to separate internal information flows based on how they are presented to the user [33]. Using the MVC pattern to interface different parts of the CropSight system, not only source code can be reused for both device-side and server-side software implementation, we could also enable modulated parallel software development to add new functions, while biological experiments were still ongoing (Fig. 2D).

To enable data standardization and integration, a RESTful API was implemented that accepts image- and sensor-based datasets and IoT device status updates in JavaScript object notation (JSON) format. All interactions between devices and the server are authenticated using a pre-shared key pair to ensure that data collection is accomplished from a trusted source. The RESTful design allows all data requested for transaction to be contained within a single request, which compiles all information into one JSON object and then transmits through an HTTP POST request. The *Model* implementation allows us to determine dynamic data structures, as well as to manage logic and rules of the CropSight system. The entity–relationship model (ER diagram) used for establishing the database, including entity types and specifies relationships between the entity types, can be seen in Supplementary Fig. S4.

Based on the PHP server (Apache tested) and the SQL server (MySQL and MariaDB tested), the *Controller* component responds to user input and internal interactions on the data model. The controller receives image, sensor and system status as the input data flows, validates them, and then passes them to the model component, first on a distributed device-side server and then transmitted to a globally accessible server-side server, which mirrors the input data. Internet connections are required, if the input datasets need to be transferred from a field experiment site to onsite servers. The form of data transmission can be either wired ethernet or WiFi network. The *Controller* administers data collation between device-side and server-side by mimicking the device API call to the higher-level server API at the time that device request is programmed.

The *View* component presents the data model and user interactions in two formats. First, through an active HTTP connection and D3.js graphing engine [33], users can access distributed IoT devices via web browsers (Chrome and Firefox tested) installed on any smart device in the field or in greenhouses. The device-side CropSight provides a tailored GUI window, within which users can deploy (see Additional File 1), monitor, assess, and download captured data on demand. Second, the device-side system synchronizes with the server at regular intervals, based on which CropSight provides a more comprehensive GUI to present both experimental and technical status (i.e., system status) of ongoing experiments. The device-side system is designed to be distributed. So, if a given IoT device cannot make a direct Internet connection for any reasons, the device-side system will enable local data storage as a server node. After the networking is re-established, the system can then forward collected data automatically (the onboard USB memory stick can store up to 60 days’ of image and sensor data).

### Experiment and data management

Monitoring dynamic plant phenotypes such as height, growth rate, growth stages, and associated climate conditions in biological experiments can be a laborious and time-consuming task. It is even more challenging if we need to calibrate and verify datasets collected from sensing devices deployed in different sites. In particular, low-quality and missing data often lead to analysis errors and unusable results, which normally can only be identified after the completion of experiments [34]. Hence, the server-side CropSight system was designed to oversee ongoing experiments based on representative daily images, hourly sensor data collected from each phenotyping device, as well as experimental settings such as genotype, treatment, drilling date, plot position and biological replicate.

**Figure 3:**
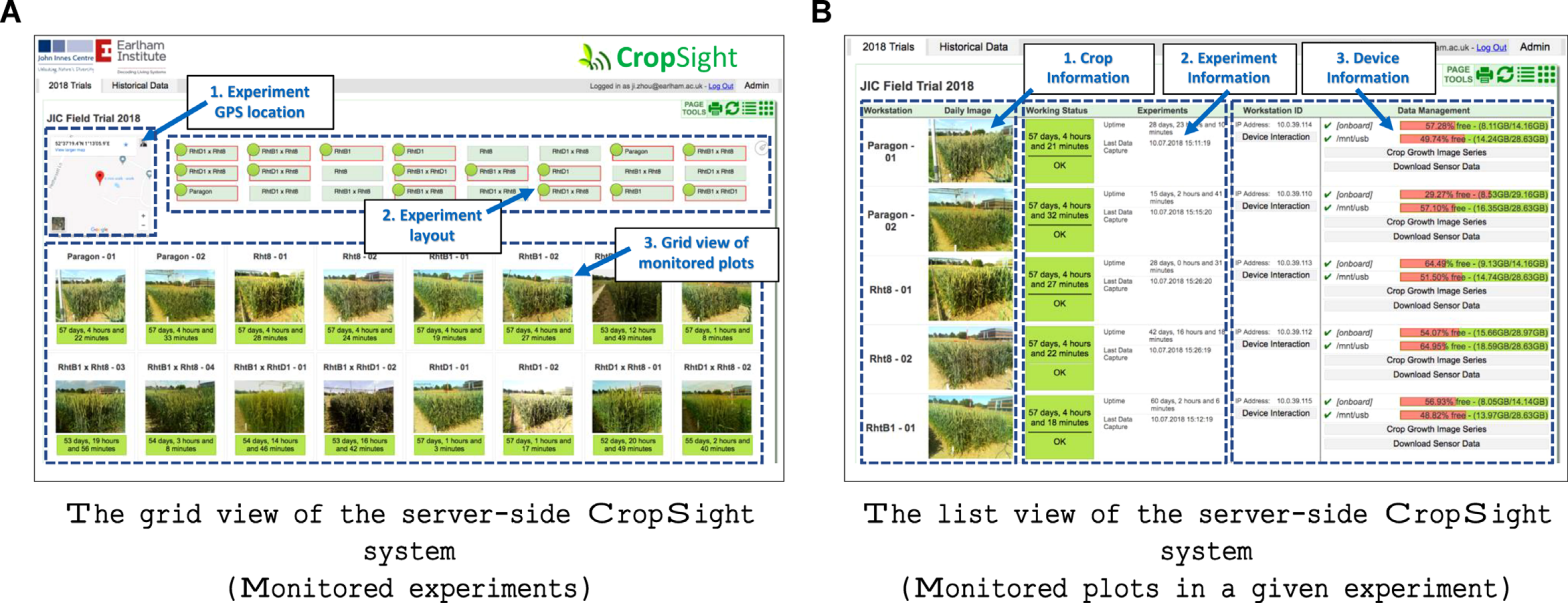
System views of the server-side CropSight system. **(A)** The user interface is accessible through a web browser on any computing device. The grid view of the system is designed to present all experiments, including geolocation of the experiments, their experimental layouts, monitored plots and genotypes, experiment duration, and representative daily images. **(B)** The list view shows detailed statistics of all monitored crops in a given experiment, including crop information (genotypes and daily images), experimental information, and distributed phenotyping information such as workstation ID, storage, IP address, image and sensor data download, and device interaction functions via flask-based HTTP interface.

**Figure 4:**
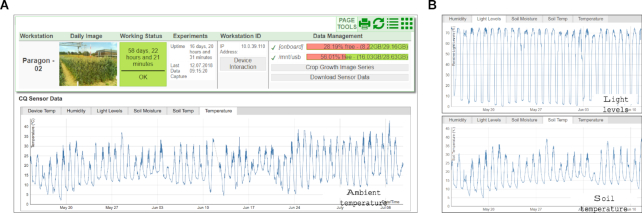
The individual view of the server-side CropSight system. **(A)** The individual view of the server-side CropSight system monitoring crops in the field, detailing device and experiment information together with captured microclimate data. **(B)** Web-based graph visualization of hourly sensor readings during a given experiment, showing ambient temperature, ambient humidity, field lighting, soil moisture, and soil temperature variation in the plot region.

The interfaces of experiment and data management are presented in Fig. 3, which integrate experiment location, plot map, and crop/experiment/device information to enable quick cross-referencing so that crop management decisions can be made while experiments are ongoing. As shown in Fig. 3A, for a given experiment, the grid view provides global positioning system (GPS)-tagged project geolocation, identifiers of installed phenotyping devices, representative daily images of monitored plots, and color-coded status indicator showing the operation mode of each distributed device. CropSight reads the device-side server's GPS coordinates and presents the geolocation in an embedded Google Map for users to locate the experiment. In addition to the GPS location, an embedded plot map is also provided demonstrating the position of each monitored plot or pot in the field or in greenhouses together with color-coded status markers, indicating whether extra attention is needed (e.g., green for operating, amber for idle, and red for device termination or operational error). These markers in the plot map can be clicked, which will bring the user to the detailed view of an individual device (Fig. 4). Each distributed phenotyping device uploads a daily representative image of the monitored plot or pot. The resolution of the image is 640 × 480 pixels, downsized from 2,592 × 1,944 pixels to enable effective data transmission for large-scale device-server data synchronization. The image is automatically selected based on file size, intensity, and image clarity. Image calibration and white balance for infield crop imaging are accomplished via phenotyping devices such as CropQuant workstations [24]. The automated adjustment of white balance gains and exposure mode under changeable lighting conditions are included in the Python script available in the CropSight project repository on GitHub ([34], Assets Section, camera_capture_script.py).

The grid view of these representative images is used as a snapshot of the experiment, so that users can quickly assess plant growth and performance of each genotype without regularly walking in the field during the growing season. We have developed an image analysis algorithm to automatically select high-quality images from daily image series to reduce manual interference on operating phenotyping workstations [35], as well as a number of Python-based software such as Leaf-GP to analyze growth phenotypes [36]. However, to maintain the independence of CropSight, these algorithms have not been integrated in the infrastructure.

The list view provides a table of status that incorporates crop information with experiment and device details (Fig. 3B). This view is mainly used for project maintenance proposes, which contains three sections. First, similar to the grid view, crop information identifier lists phenotyping devices installed in the experiment. Second, experiment information includes a colored status indicator to display the operational mode of a given device, the experiment duration of a given device, and the latest time stamp of data synchronization. Device uptime (i.e., experiment duration) is computed using the device's internal clock (i.e., the Linux uptime command) and the time when the latest image is captured. Third, distributed device information shows: (1) each device's onboard storage, using filled bars to indicate the percentage of space left in gigabytes based on regular 30-minute updates; (2) buttons to download image- (i.e., “Crop Growth Image Series,” in monthly Zip archives) and sensor-based (i.e., “Download Sensor Data,” in a comma-separated value file) datasets collated during the experiment from the SQL database; and (3) device interaction buttons, providing direct device control and remote system configuration via Secure Shell or VNC.

### Continuous microclimate visualization

Microclimate is important evidence for plant scientist to monitor radiation/ambient/soil variation in different locations over the whole experiment site, an important factor that closely connects with the performance at both plant and plot levels [37]. To facilitate the monitoring of microclimate during an experiment, a comprehensive visualization function has been developed for CropSight (Fig. 4). By accessing a given phenotyping device's detail page, collected environmental factors can be viewed as individual line charts along with the device information. IoT-based climate sensor readings are logged with the central server and then indexed by device and location, allowing near real-time microclimate readings (30-minute updates) of monitored regions. The visualization is done in the web browser using the D3 JavaScript library. In our case, we can soundly retrieve readings such as device temperature (to assess device performance), ambient relative humidity, ambient temperature (Fig. 4A), light levels (based on light intensity), soil temperature, and soil moisture (Fig. 4B). The microclimate datasets acquired from multiple locations across the field can also be used for data calibration to generate a normalized and highly reliable environmental reading of the experimental site. The CropSight system accepts collective readings from most off-the-shelf climate sensors and hence is open to the expansion of new environmental variables. The environmental sensors used in our experiments are: DHT22 digital temperature and humidity sensor, TSL2561 luminosity sensor, DS18B20 waterproof digital temperature probe, and analogue capacitive soil moisture sensor. Ambient temperature and humidity sensors were incorporated into the housing of the phenotyping workstations, and soil sensors were inserted into the ground of the plot, attached to the phenotyping workstations via cables.

### Applications in wheat field experiments

**Figure 5:**
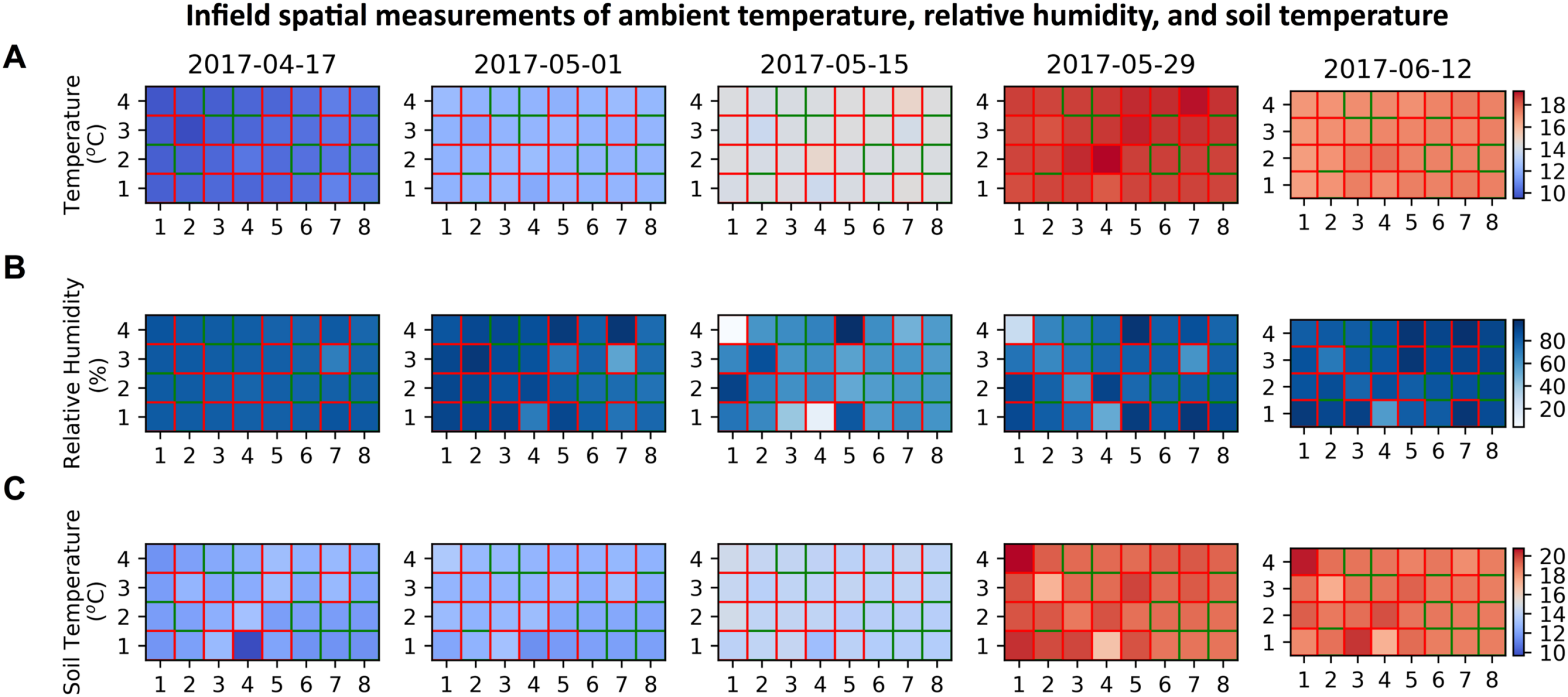
Infield spatial measurements of microclimate conditions collated by the CropSight system. **(A, B)** A heat map of ambient sensor reading of temperature and relative humidity recorded during the growing season. Each cell represents a plot in the 2017 field experiment. Real sensor reading outlined in red and interpolated values outlined in green. **(C)** A heat map of soil-based sensor reading of soil temperature recorded during the growing season.

A key element of modern agriculture is to closely monitor dynamic crop performance and agricultural conditions to predict and plan crop production [38]. Plant breeding and GxE studies also rely on high-quality and high-frequency crop-environment data to produce accurate growth models for yield and quality prediction [39, 40]. The CropSight system provides users with quick access to environmental factors recorded by each distributed phenotyping device during the growing season. Based on the position of a given phenotyping device, seasonal microclimate datasets can jointly form a dynamic growth condition map showing environmental conditions and variance in the field (Fig. 5).

In a 253-day field experiment of 32 wheat genotypes within the single genetic background of Paragon (a UK spring wheat variety) completed in 2017, we installed 16 CropQuant field phenotyping workstations to monitor 6-meter wheat plots to collect continuous crop growth image series as well as associated microclimate conditions such as ambient temperature, relative humidity, light levels, soil temperature, and soil humidity. When the datasets were being collated in CropSight, a field map of dynamic microclimate conditions at key growth stages (i.e., from early booting to early grain filling, 56 days) was gradually produced, showing the increase in ambient temperature (Fig. 5A), the variation of ambient moisture levels (Fig. 5B), and the steady increase in soil temperature (Fig. 5C). To simplify the presentation, the microclimate heat map was presented with data at 14-day intervals, where wheat plots installed with sensors were outlined with red and plots without sensors were outlined with green, where climate data was produced through data interpolation methods based on adjacent readings (Fig. 5). The period of the interval can be flexibly changed, and the microclimate readings are retrievable as soon as data synchronization is finished (Supplementary Fig. S5 and Additional File 2). Furthermore, the climate datasets can be used for cross-validating the soundness of infield sensors, e.g., whether soil temperature correlates with ambient temperature (Supplementary Fig. S5A) and why readings from many low-cost sensors could provide more representative information of the field in comparison with one expensive central weather station (Supplementary Fig. S5B).

Utilizing this approach, dynamic environmental conditions throughout the field can be recorded with very low-cost climate sensors, which can then be scaled up through interpolation methods to cover regions without sensors. To soundly interpolate environmental data, the placement of climate sensors needs to be standardized to ensure effective data coverage. Depending on measurement requirements, standards for sensor placement can be based on the estimation of evapotranspiration [41]. Through our wheat field experiments between 2016 and 2018 at Norwich Research Park in the United Kingdom, combining distributed sensors and the CropSight system was capable of providing high-quality crop performance and growing conditions datasets for our changeable experiment needs.

### Comparison between multi-year experiments

**Figure 6:**
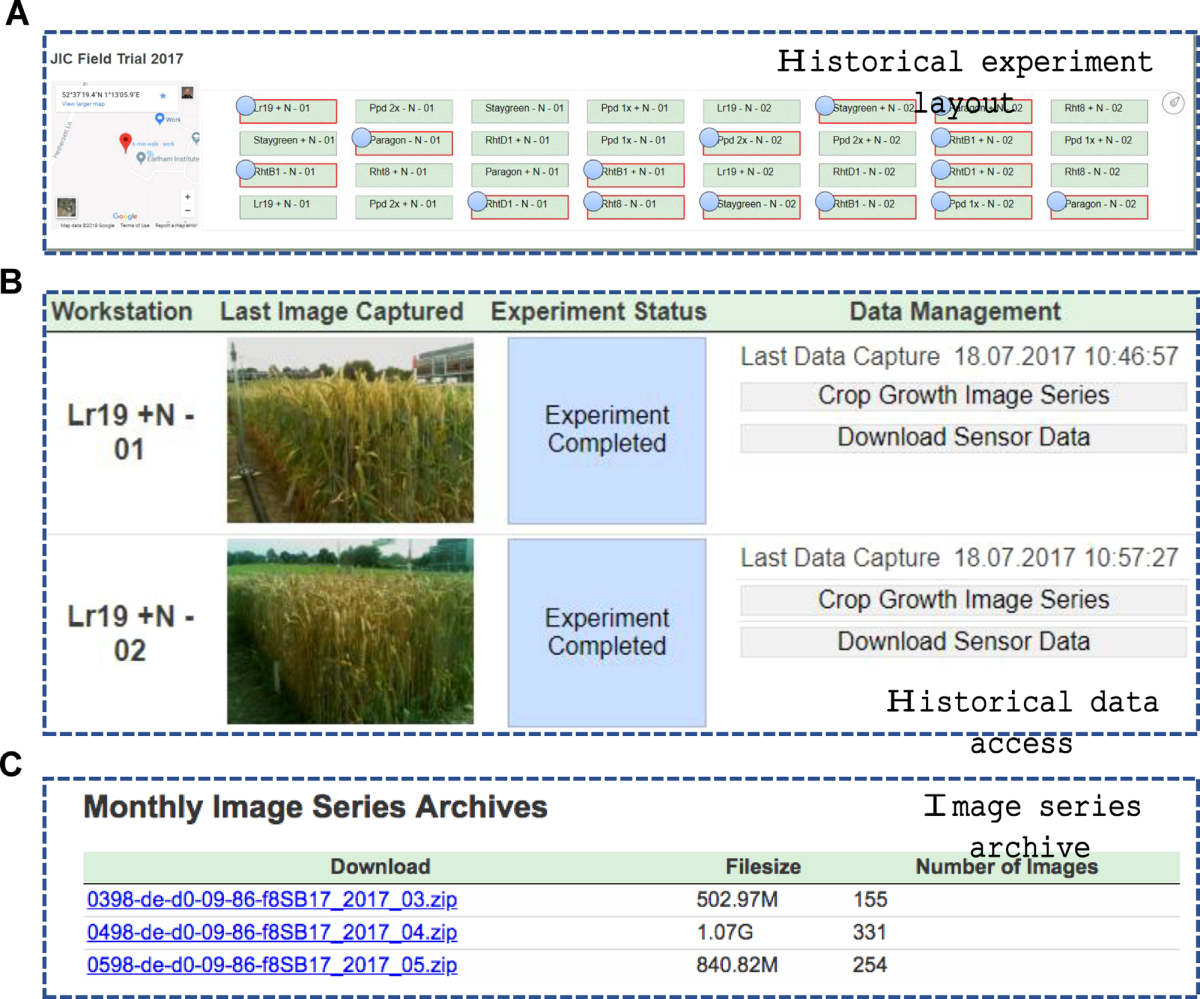
Historical experiments and data access. **(A)** The CropSight system provides access to historical experimental datasets, including the geolocation of all experiments as well as all plots monitored in a completed experiment. **(B)** In a completed experiment, the last image captured in the experiment and historical image- and sensor-based data can be downloaded. **(C)** The download links for monthly image series archived in cloud.

CropSight not only provides tools for monitoring ongoing infield and indoor experiments but also supplies toolkits to reference and download historical datasets. An important part in crop research is being able to compare collected results with past experiments. To this end, CropSight stores all image and sensor data and manages these historical datasets with easy reference and access (Fig. 6). Historical datasets can be retrieved through the frontpage, similar to ongoing experiments (multiple projects can be administered by CropSight simultaneously). After opening a completed project, users can display the GPS-tagged geolocation of an completed project and devices used in the project together with project references (Fig. 6A). By clicking a specific plot within the experimental field, CropSight can directly reference environmental and image datasets in the plot, with device name, date of last capture, and last image taken by the phenotyping device (Fig. 6B). If users want to revisit previous datasets in the project, they can download both sensor data packages and/or growth image series in monthly archives by clicking the archive links (Fig. 6C). This design enables a unified cloud-ready platform to facilitate both ongoing and historical data management for in- and post-experiment comparison.

## Discussion and Outlook

The continuing challenge of global food security caused by fluctuating environments and a narrower range of genetic variation of modern crops requires innovative thoughts and technologies to improve crop productivity and sustainability [2, 42, 43]. As European infrastructures for sustainable agriculture (e.g., EMPHASIS and Analysis and Experimentation on Ecosystems) have identified, openly shareable solutions built on widely accessible digital infrastructures are likely to provide an effective solution to address the challenge by integrating novel scientific concepts, sensors, and models [44, 45]. The CropSight system presented here is scalable and open source, providing the scientific community a number of interfacing options to adopt and extend. The openly available platform integrates high-frequency environmental data and crop images automatically, which can be used to enable both phenotypic analyses and agricultural decision making. By associating environmental conditions with crop growth data, we also trust that the system is capable of forming a sound base for reliable GxE studies. More importantly, CropSight provides geolocation and remote sensor readings of current and historical experiments, a comprehensive solution to enable multi-site and multi-year cross-referencing of crop performance and growth conditions.

Because CropSight facilitates the real-time access of microclimate conditions and crop imagery (through live video streaming) in the field or in greenhouses, either through a smart device or an office PC, users can make a quick decision of crop performance, growth stages, and plot conditions of any monitored location distributed in a given experiment, field, or site. Furthermore, automatic data transmission allows for centralized data and experiment management, which means that the system can be scaled up to the national scale if a broader IoT in agriculture infrastructure is in place. As collected data is annotated and pre-selected on distributed phenotyping or IoT devices, only standardized crop-environment datasets are collated to support detailed traits analyses and cross-referencing. Finally, openly sharing results from different sites and different experiments will enable crop researchers, breeders, and farmers to gain great benefits, e.g., predicting and prewarning disease spread at the national scale so that early adoption of preventative measures can be arranged.

Presently, many governments are shifting their focus toward innovative technologies to modernize crop and agricultural research. The UK government, for instance, has invested heavily in IoT-based technologies to address challenges on yield production, food traceability, environmental issues, incompatibility, and lack of infrastructure [46]. We believe that CropSight can address some of the current challenges directly. For example, logging historical data and annotating crop growth and environmental effects within monitored fields can increase crop traceability. To reduce the overall use of agrochemicals as part of a precision farming strategy [47, 48], CropSight can be used to identify the appropriate timing and areas for chemical application together with infield imaging and ambient sensors. Water is in limited supply for large regions of the globe, and the reduction of unnecessary irrigation would be of large benefit to the cost-effectiveness of agriculture [49, 50]. As discussed previously, CropSight is built with near real-time environment monitoring mechanisms including soil temperature, soil moisture levels, and ambient humidity. Hence, it can provide information crucial to making decisions and targeting irrigation as they relate to timing and location. Additionally, by linking extra climate sensors with IoT devices, further environmental readings can be extended in CropSight for growing agricultural needs.

Besides environmental and crop growth monitoring, historic and current datasets collated in a central system can also deliver predictive powers. An example of potentially predictable situations is the “Smith Period” for predicting late blight in potato crops [51]. Late blight is shown to be likely to occur during a “Smith Period,” which is defined by a period of two or more days with a minimum temperature of 10°C and humidity of 90% or above for at least 11 hours in each day. Having direct access to dynamic sensor readings on the CropSight can make the monitoring of specific environmental patterns much easier and thus establish an important tool to inform farmers and growers to apply fungicides and chemical treatments to the appropriate areas. Hence, CropSight has a high potential to serve sustainable agriculture and environmental friendliness of food production under today's changing climates.

### Future development

To establish a data and experiment information management system that is scalable and usable on regional, national, or even global crop research and agricultural practices, we believe that with further development, CropSight in connection with distributed IoT sensors can meet the future demand of usability and scalability. One area of expansion is in scalability. The system is currently tested on a local server with a direct network connection to at least one of the distributed nodes. To allow the expansion at a larger, national, or even global scale, the reliance on maintained servers would be less effective than a true cloud-based service. Hence, moving the CropSight system to a globally accessible cloud server with cloud-enabled distributed storage is a potentially feasible approach that removes the requirements for institutions and agricultural practitioners to maintain servers and storage. Given the lack of network infrastructure in rural areas in many countries, the addition of 3G or 4G mobile data networks to key distributed nodes in the field can improve the infield network, upon which the data communication of a large number of agri-tech devices can be relied.

Another prohibitive factor in IoT in agriculture is the quantity and costs of IoT devices required to cover an entire field. Based on our three-year field experiments, we believe that installing sensors and phenotyping workstations to cover every area in the field is unnecessary. Figure 5 shows that the data interpolation approach that is applied can generate microclimate readings between randomly positioned stations to model environmental variation across the whole field. This subsampling approach has produced high-quality environmental readings, which could be used to improve the effectiveness of IoT applications in agriculture. Additionally, with the development of national IoT infrastructure, the similar subsampling idea can be expanded to a larger and multi-site level, which can then truly help inform decision in crop research and agricultural practices across a country's arable land.

## Availability of source code and requirements

Project name: CropSight for wheat prebreeding in Designing Future Wheat

Project home page: https://github.com/Crop-Phenomics-Group/cropsight/releases [35]

Operating system(s): Platform independent

Programming language: Python, PHP, JavaScript, SQL

Requirements: Apache (or other PHP5+) server, MySQL (or other SQL) server, a recent version of Chrome, Firefox, or Safari

License: BSD-3-Clause available at https://opensource.org/licenses/BSD-3-Clause

RRID:SCR_016870

## Availability of supporting data

The datasets supporting the results presented here are available at the CropSight Project page [35]. Snapshots of source code and other supporting data are also openly available in the GitHub repository [35] and *GigaScience* database, GigaDB [52].

## Supplementary Material

GIGA-D-18-00414_Original_Submission.pdf

GIGA-D-18-00414_Revision_1.pdf

Response_to_Reviewer_Comments_Original_Submission.pdf

Reviewer_1_Report_Original_Submission -- Andri Nugroho 11/19/2018 Reviewed

Reviewer_2_Report_Original_Submission -- Bing Lu 11/20/2018 Reviewed

Reviewer_2_Report_Revision_1 -- Bing Lu 12/21/2018 Reviewed

Supplemental Files

## Additional files

Additional File 1.docx (CropSight Installation Instructions and Interface Details)

Additional file gives step-by-step instructions for initialising the system through an existing PHP webserver with SQL database, details of RESTful API required fields necessary for device interaction, and addition detail of distributed installation and database integration.

Additional File 2.html (Algorithm to generate plotted figures)

Additional file contains full python code to replicate plotted figures within the paper, displayed within an exported iPython notebook. All datasets shown within the plotted figures of the paper are available at the project GitHub repository.

**Supplementary Figure S1**. The device-side CropSight system login GUI and remote system configuration. **(A)** The device-side CropSight system gives GUI-based access to each phenotyping device's user interface. **(B)** The system allows device management and remote system configuration such as live video streaming to assist in calibration and experiment setup.

**Supplementary Figure S2**. Archived image- and sensor-based experimental data access. (**A&B**) Archived data access of 2016 and 2017 wheat field experiments, allowing browsing and downloading of previously completed infield experiments. **(C)** Accessing multiple indoor and infield experiments and archived historical data to enable cross-referencing crop growth and environmental conditions.

**Supplementary Figure S3**. The network topology of self-operating crop phenotyping in the field. A number of nodes form a star network with a central in-field server node, which communicates with the CropSight system through an in-field wireless network.

**Supplementary Figure S4**. Database Entity-Relationship diagram detailing high-level entities within the CropSight database and the relational links between primary, composite and foreign key fields. The ER diagram also describes the structure of database tables, omitting simple storage fields.

**Supplementary Figure S5**. Validating climate sensors deployed in the field. (**A**) The cross-validation of two different sets of sensors, normalised soil and ambient temperature readings. **(B)** Different reading between distributed ambient humidity sensors (15 placed in the field) in comparison with a central weather station, showing different climate readings.

## Abbreviations

API: application programming interface; ER: entity relationship; GPS: global positioning system; GUI: graphical user interface; GxE: genotype by environment; HTTP: hypertext transfer protocol; IoT: Internet of Things; JSON: JavaScript object notation; MVC: model-view controller; PHIS: phenotyping hybrid information system; PHP: PHP hypertext pre-processor; SQL: structured query language; USB: universal serial bus; VNC: virtual network computing; WSN: wireless sensor network.
