# Supplementary material for: CropSight: a scalable and open-source information management system for distributed plant phenotyping and IoT-based crop management
Source: Gigascience. 2019 Jan 31;8(3):giz009. doi: 10.1093/gigascience/giz009 (PMC6423370; doi:10.1093/gigascience/giz009)
Supplement: Supplemental Files [file giz009_supplemental_files.zip › Additional File 1.docx]

#### **CropSight Installation Instructions and Interface Details**

#### **Database Initialisation**

- 1. A user must be created on the SQL server with the username ‘cropmonitor’ and a secure password which must be recorded.
  2. A database named ‘cropmonitor’ must be created on the SQL server and read/write access given to the CropSight user
  3. In the supplied source code is the SQL file /sql/database.sql, this file must be run on the CropSight database using either an SQL admin tool such as phpmyadmin or the SQL command: source /pathtosource/sql/database.sql. This will initialise all database tables.
  4. Due to security restrictions for sensitive data, CropSight accounts cannot be created through the interface. To add a user account, manually add the users name and email address to the table cropmonitor.users. Leaving the password blank will prompt the user to set a password on first login.

#### **PHP Initialization**

- 1. Edit the file in the supplied source code /php/database.php to add the password created in Database Initialization Step 1 to the empty field $sql_password.
  2. Copy all files in the php folder (not including the folder itself) to the base php folder of the webserver. For example, on a standard Linux Apache server this folder would be /var/www/html.
  3. Connect to the webserver using it’s IP or web address, successful installation will show the CropSight login screen. This can be logged in using the user account created in Database Initialisation Step 4.

#### **RESTful API**

To enable data integration and standardization, CM implements a RESTful API which accepts data and status updates from devices in JSON format. All interactions between devices and the server are authenticated using a pre-shared key pair ensuring data is collected from a trusted source.

The RESTful design strategy ensures that all data required for a transaction is contained within a single request, allowing devices to compile all information into one JSON object and transmitted through an HTTP POST request. Required API fields are:

| ***server key:*** | A pair of pre-shared 24 character randomly generated strings for authentication |
| --- | --- |
| ***device id:*** | Unique device identifier (MAC address + project ID currently used) |

Required API fields for status update are:

| ***uptime:*** | Device active time in seconds |
| --- | --- |
| ***lastdatacapture:*** | Date and time of last image captured |
| ***hostname:*** | Device’s human identifiable name |
| ***storage:*** | JSON array of storage objects containing total and available size (in bytes) and device mountpoint |
| ***duration:*** | Expected duration before next status update (in minutes) |

#### **Distributed Installation**

One of the key features of CropSight’s design in distributed installation. The system can be installed on any platform which supports an appropriate PHP server (Apache tested) and SQL server (MySQL and MariaDB tested). For in-field installations, the system has been loaded onto a Raspberry Pi single board computer and installed alongside phenotyping devices. With an active internet connection, data collated by the distributed server is also transmitted offsite to a globally accessible server which mirrors the information and data captured for remote access. Internet connections required can be in the form of wired ethernet, Wi-Fi or 3G/4G mobile data. Collation is performed by mimicking the device API call to the higher-level server after any data storage had been performed, at the time of device request.

#### **Database Integration**

The database used to store collected phenotypic and environmental data is SQL based. Supplementary Fig. 4 shows an entity-relationship diagram detailing the formation of database tables and relationships. Each device is uniquely identified by a combination of the network interface MAC address and the current project ID (set in the device’s own configuration interface). This ensures that while devices can be easily identified, the same device can be reused for separate trials without historical data being combined with current data or overwritten by new status updates.
