# Supplementary material for: CropSight: a scalable and open-source information management system for distributed plant phenotyping and IoT-based crop management
Source: Gigascience. 2019 Jan 31;8(3):giz009. doi: 10.1093/gigascience/giz009 (PMC6423370; doi:10.1093/gigascience/giz009)
Supplement: Supplemental Files [file giz009_supplemental_files.zip › Supplementary Figure 3.pptx]

## Slide 1
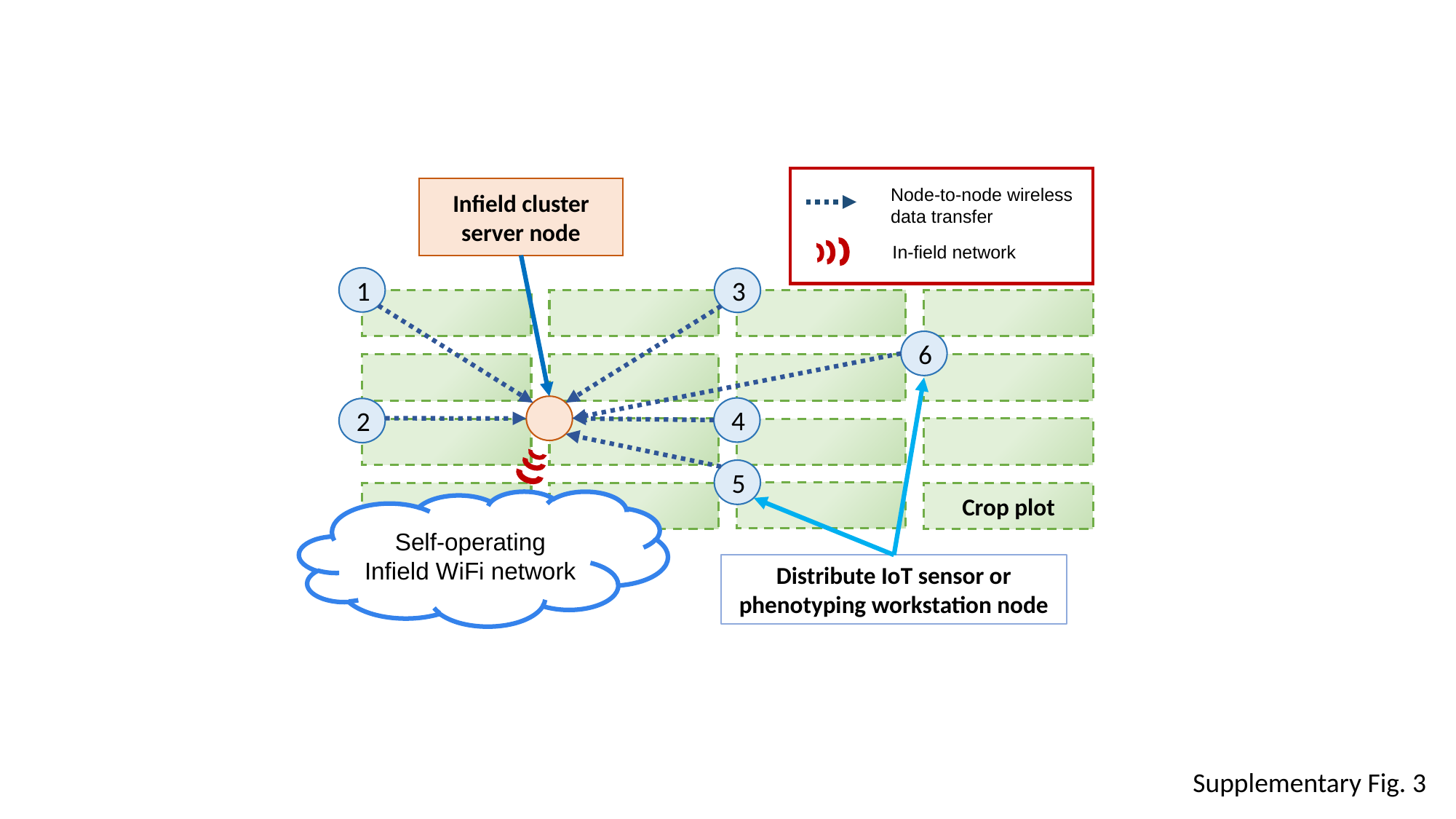

Node-to-node wireless data transfer
Infield cluster server node
In-field network
1
3
Crop plot
6
4
2
5
Self-operating Infield WiFi network
Distribute IoT sensor or phenotyping workstation node
Supplementary Fig. 3
