# Supplementary material for: CropSight: a scalable and open-source information management system for distributed plant phenotyping and IoT-based crop management
Source: Gigascience. 2019 Jan 31;8(3):giz009. doi: 10.1093/gigascience/giz009 (PMC6423370; doi:10.1093/gigascience/giz009)
Supplement: Supplemental Files [file giz009_supplemental_files.zip › Supplementary Figure 5.pptx]

## Slide 1
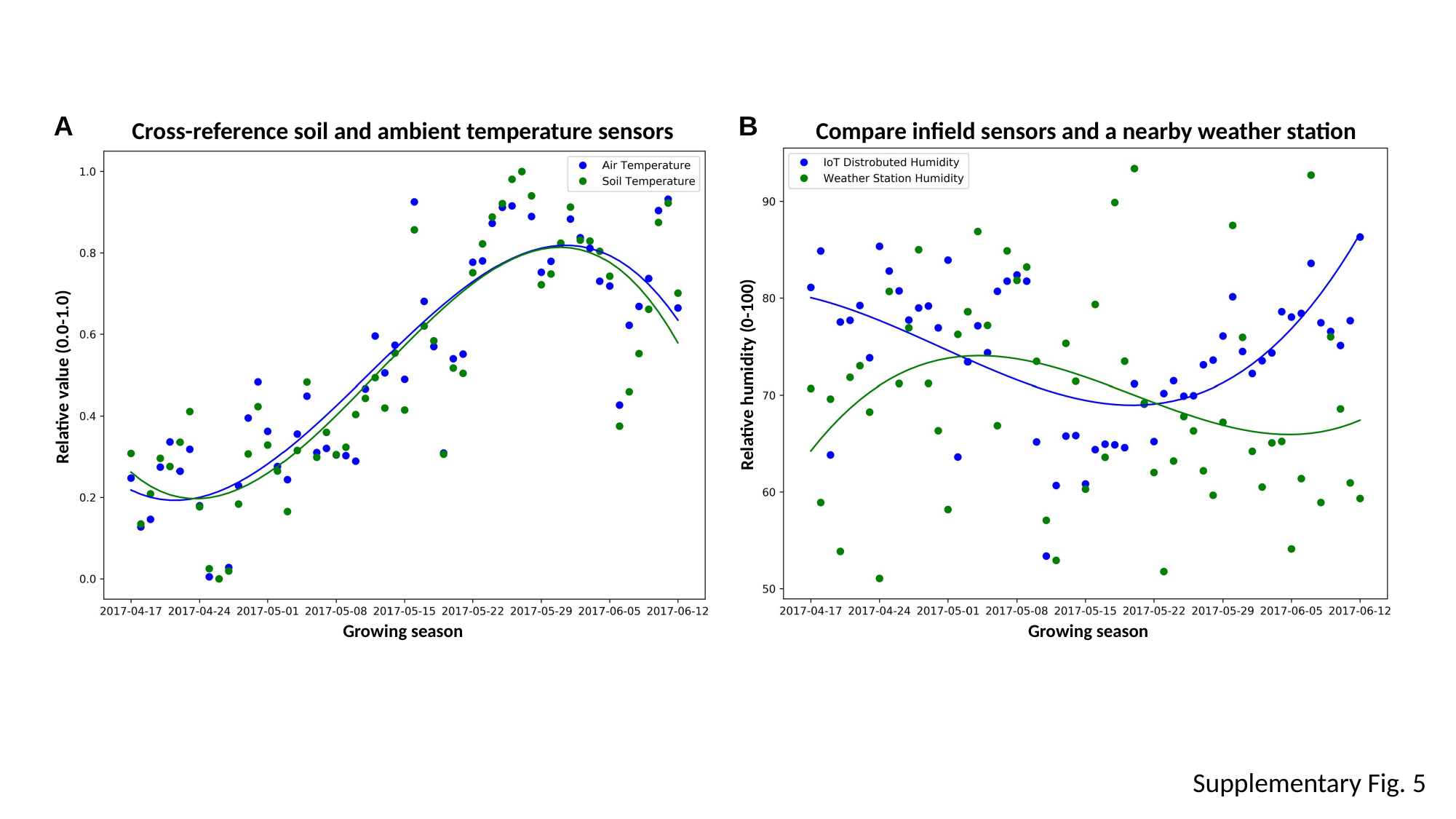

A
B
Cross-reference soil and ambient temperature sensors
Compare infield sensors and a nearby weather station
Relative humidity (0-100)
Relative value (0.0-1.0)
Growing season
Growing season
Supplementary Fig. 5
