# Supplementary figures and images for: CropSight: a scalable and open-source information management system for distributed plant phenotyping and IoT-based crop management
Source: Gigascience. 2019 Jan 31;8(3):giz009. doi: 10.1093/gigascience/giz009 (PMC6423370; doi:10.1093/gigascience/giz009)

## Slide 1
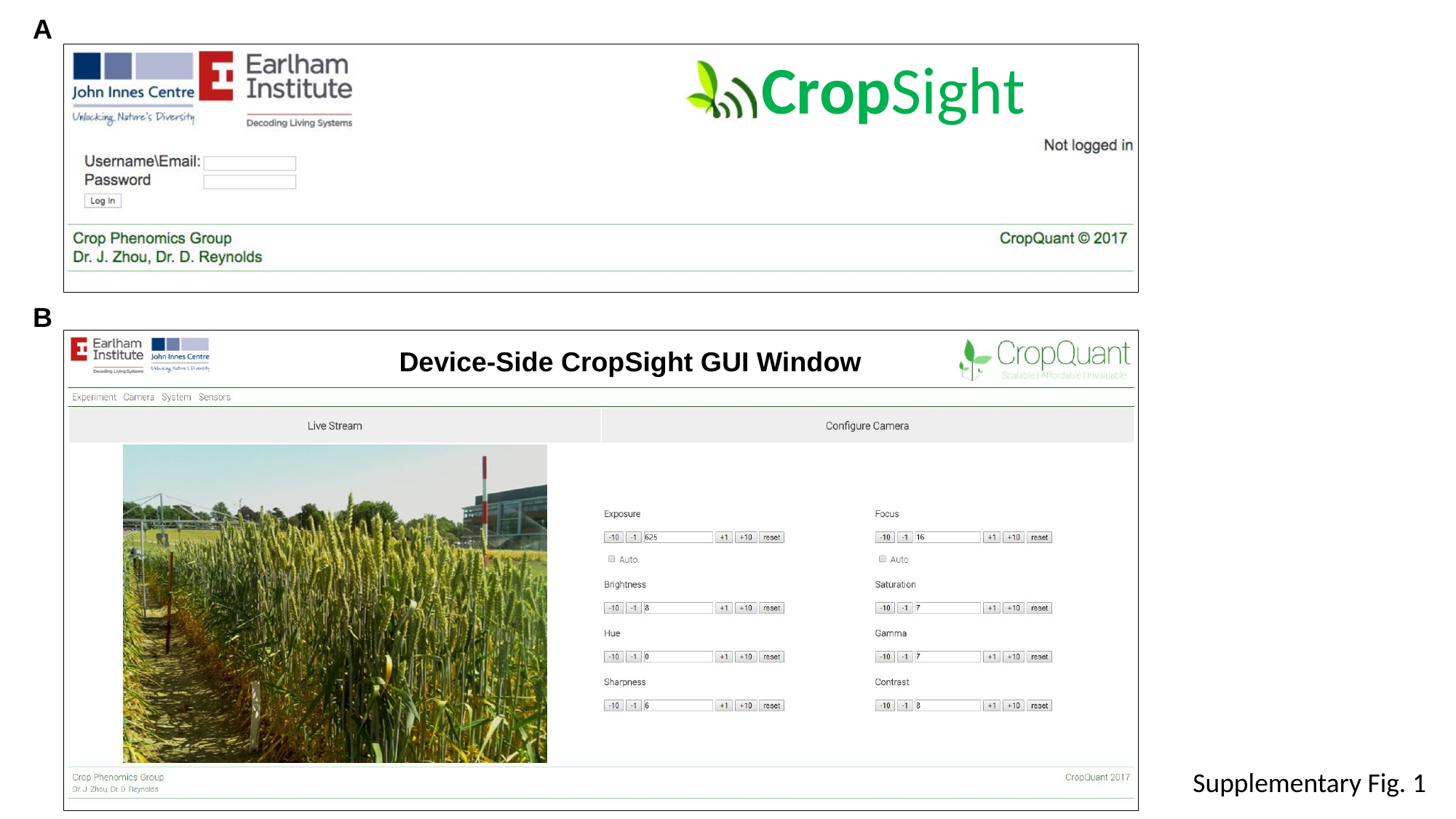

A
CropSight
B
Device-Side CropSight GUI Window
Supplementary Fig. 1

Supplement: Supplemental Files [file giz009_supplemental_files.zip › Supplementary Figure 1.pptx]

## Slide 1
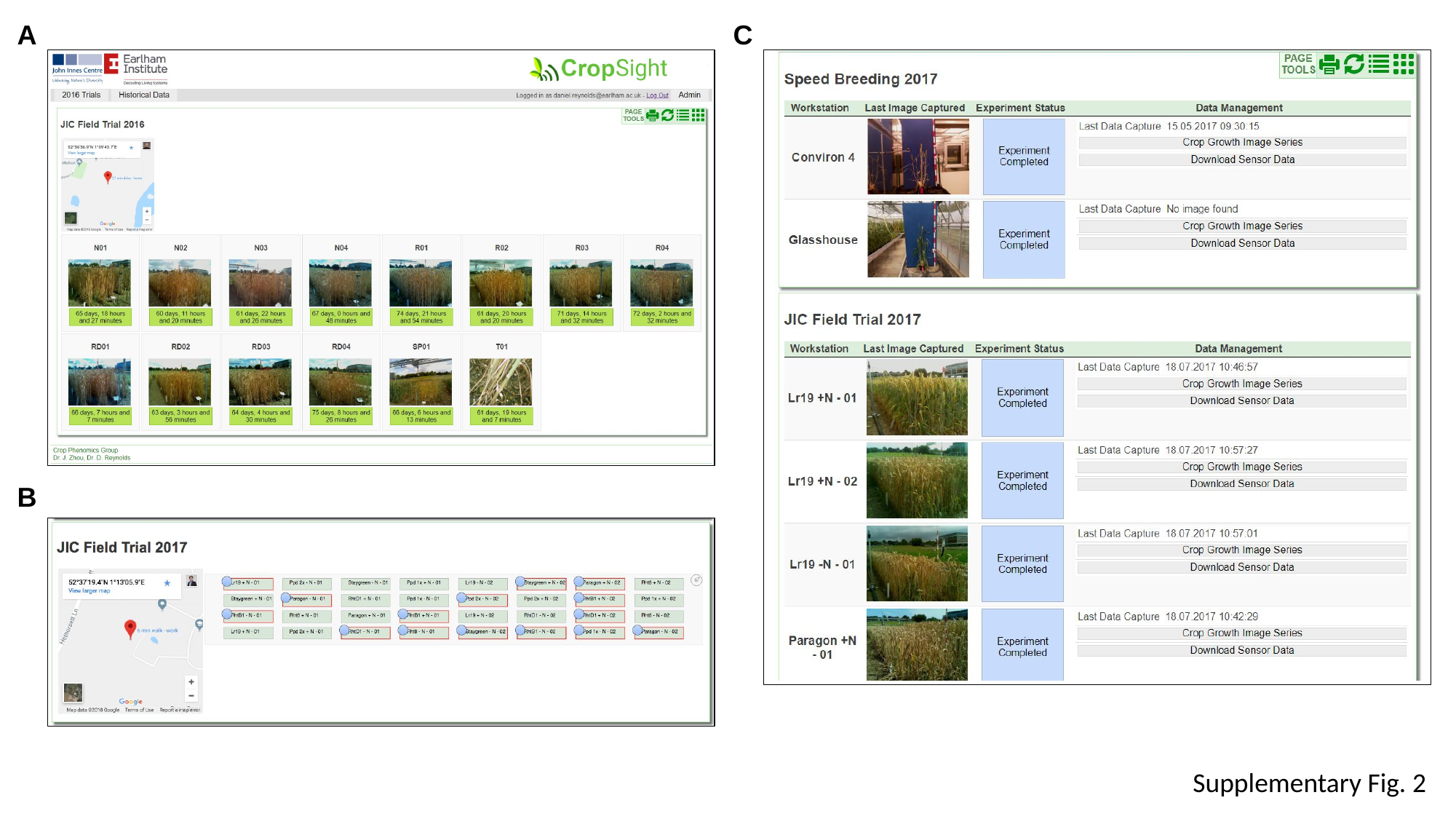

A
C
B
Supplementary Fig. 2

Supplement: Supplemental Files [file giz009_supplemental_files.zip › Supplementary Figure 2.pptx]

## Slide 1
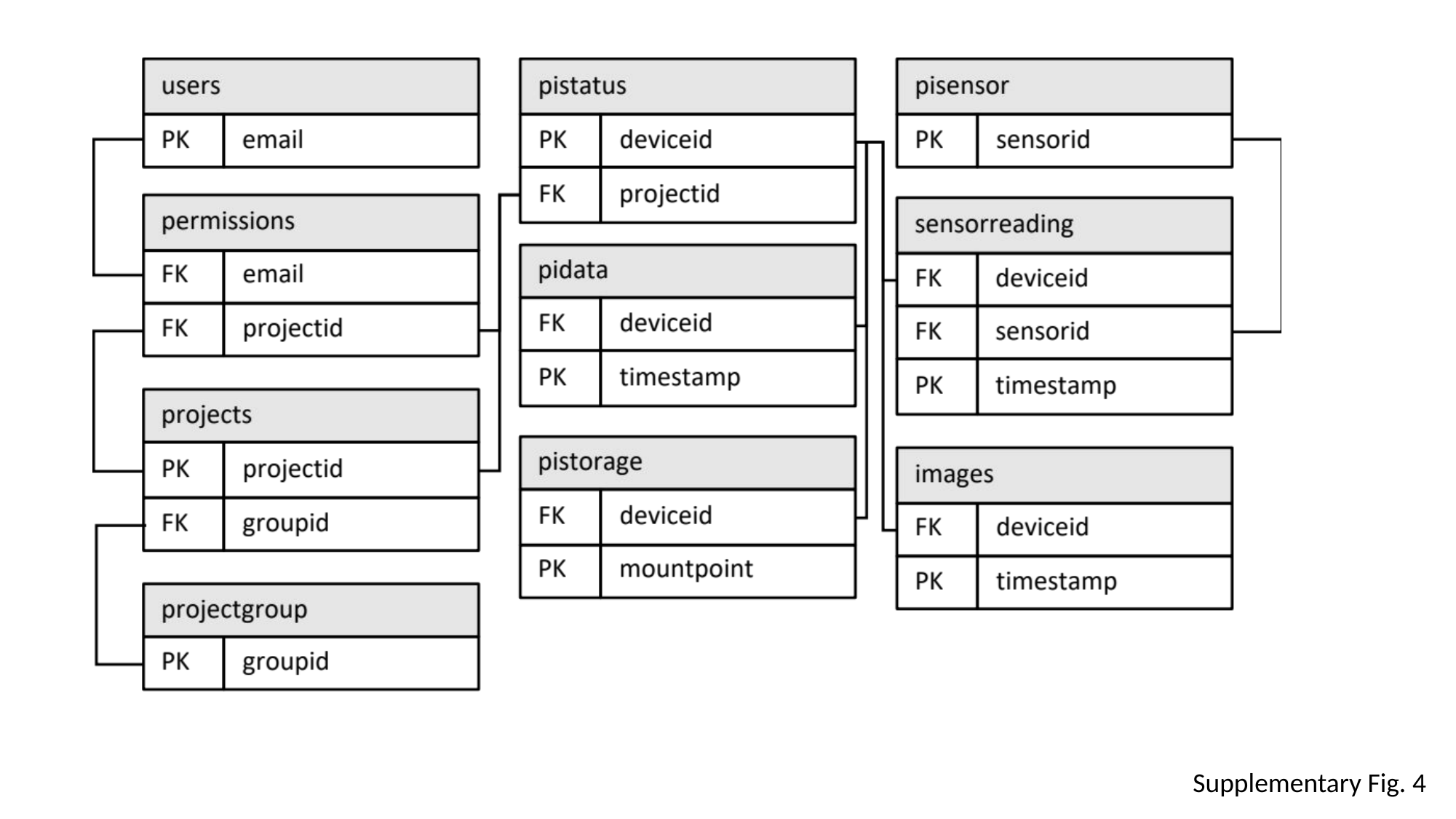

Supplementary Fig. 4

Supplement: Supplemental Files [file giz009_supplemental_files.zip › Supplementary Figure 4.pptx]
