# Supplementary material for: CropSight: a scalable and open-source information management system for distributed plant phenotyping and IoT-based crop management
Source: Gigascience. 2019 Jan 31;8(3):giz009. doi: 10.1093/gigascience/giz009 (PMC6423370; doi:10.1093/gigascience/giz009)
Supplement: Response_to_Reviewer_Comments_Original_Submission.pdf [file giz009_response_to_reviewer_comments_original_submission.pdf]

Author's Response To Reviewer Comments

Close

Dear Editor,

We thank you and the reviewers for your time and comments, which helped us improve our manuscript entitled “CropSight: a scalable and open-source information management system for distributed plant phenotyping and IoT-based crop management”. We have to change the title from CropMonitor to CropSight as “CropMonitor” has been trademarked by Defra (The Department for Environment, Food & Rural Affairs, the UK government) in one of its commercial programmes. We were informed after the manuscript was uploaded to BioRXiv and hence the change.

Based on reviewers’ comments and suggestions, we have carefully revised and improved the manuscript. Now, we would like to resubmit the revised version as suggested by the editor.

All changes we have made during the revision are highlighted in the manuscript with Word’s “Tracked Changes”. The point-by-point responses to the reviewers can be seen below. We also rearranged our Github repository for CropSight to improve its accessibility for readers and potential users to download and reference, including API document, Python-based imaging script, database source code, supporting data, interface design, raw sensor data csv files, and screenshots of different experiments presented in the manuscript.

Once again, please allow us to thank you and reviewers for your positive comments and consideration!

Yours sincerely,

Dr Ji Zhou

Reviewer #1
1. The schematic system on Fig. 1, would be better if the flow of data and information provided in different arrow and colour, and the network typology should be briefly visualized.
Response:
• Fig 1 has been modified and a legend has been added to clarify data flows throughout the user-system interactions, both internally and externally.
• Supplementary Fig. 3 has been added to show the Star Network topology applied to wheat field experiment, as well as data transfer between distributed nodes and a server node.
• The Star Network topology is described in lines 185-197.

2. The flowchart (Fig.2.C) of the data transmission from each node and server would be easier to understand if it can be visualized in completed flowchart, kindly refer the example on this paper (https://doi.org/10.1016/j.compag.2016.04.025)
Response:
• Fig 2D has been improved by adding a completed section of detailed data flows.
• The paper suggested by the reviewer has now been added in the literature review as a representative research-based data management system (lines 80-85).

3. Dealing with the utilization of camera in outdoor, is there any calibration method for white balance? Because the sunlight intensity is different every sampling. If there are any method to white balance adjustment it would be more useful.
Response:
• Although the imaging function is not part of the CropSight system, infield crop growth imaging function has been described briefly in lines 260-264.
• The Python-based imaging script has also been added to the GitHub CropSight project repository for download and reference (please go to https://github.com/ Crop-Phenomics-Group/CropSight/releases/, camera_capture_script.py).

4. The environmental sensor position during environmental measurement also should be standardized, if it will be used for estimating the reference Evapotranspiration (ETo), it should follow the standard on FAO56 Penmann Monteith
Response:
• While the placement of sensors is out of the scope of this information system article as it is independent of the CropSight system, we have improved the manuscript to emphasise the importance of sensor standardisation and infield positioning in lines 299-305 and lines 334-339.

Reviewer #2
1. Line 60-93, the introduction of different platforms is good. One concern is that the remote sensing imagery has long been recognized as an essential data source for evaluating crop properties over large areas (as sensors cannot be deployed to cover large areas), how the platforms mentioned here deal with remote sensing imagery and extract crop information?
Response:
• The focus of this manuscript is researching and developing data and experiment management software systems, including image- and sensor-based data transfer, and data collation. Hence, we focused on reviewing the literatures published in the relevant research domains.
• To reflect reviewer's concerns in terms of evaluating crops over large areas using imagery sensing, we have improved the introduction section by adding new text of image-based phenotyping approaches and a new literature (lines 64-85).
• We are talking about how sensors and analysis algorithms could be utilised for dealing with larger areas and maintain quality crop information in the manuscript. To emphasise on this matter, lines 267-271 and lines 334-339 have now been added to the manuscript.

2. Line 235, the authors described uploading images of crops to server and users can check the

images to understand crop condition. Since there may be a large number of photos taken every day/week, manual evaluation would be labour intensive. Is that possible to add some software that can automatically analyze these images and provide results to the users?
Response:
• Computer-vision based algorithms developed for analysing crop growth and phenotypic analysis using crop image series are independent of the CropSight system and have been described in Zhou et al [1], which is under review at the moment.
• We followed the reviewer's comments and made clear in the text (Lines 267-271).
• The analysis algorithms are not integrated into the CropSight system, because:
a. These algorithms have been described in [1];
b. They rely on specific phenotyping devices (e.g. CropQuant workstations);
c. CropSight is platform independent, which means it is expandable to incorporate other hardware sensors and single-board computers;
d. It is beyond the scope of this open-source data/experiment information management system.

1. Zhou J, Reynolds D, Websdale D, Le Cornu T, Gonzalez-Navarro O, Lister C, et al. CropQuant: An automated and scalable field phenotyping platform for crop monitoring and trait measurements to facilitate breeding and digital agriculture. bioRxiv [Internet]. 2017;1–17. Available from: http://www.biorxiv.org/content/early/2017/07/10/161547

3. The authors introduced extensively the integration or connection of various sensors in the system, but didn't describe clearly which specific sensors can be integrated (e.g., soil moisture sensor? Fertilizer sensor?), how to setup these sensors in the field, and how the data from sensors are analyzed. These information will help readers to further understand the operation of the monitoring system.
Response:
• Lines 299-305 have now been added to specify exactly which sensors have been used in experiments and their installation in the field, together with the clarification of how the CropSight system collated data generated by these sensing modules as well as the future expansion.
• Lines 334-336 have been added to explain the sensor placement.
• Although data analysis is not within the scope of the CropSight system, we have added and described briefly the Python-based imaging script (lines 260-264), image selection (lines 267-271), and Additional File 2 (an algorithm to analyse environmental factors using plotted figures).
• All scripts described above have been added to the GitHub project repository for download and reference (https://github.com/Crop-Phenomics-Group/CropSight/ releases/).

4. In the Discussion and Outlook section, specifically 343-357, the authors discussed the potential of applying the monitoring system in real world to solve various challenges, which is good. However, the authors didn't describe clearly the challenges in deploying the system for large areas. How many sensors and how much cost needed? Although the authors indicated in 370-391 that the system is scalable and the cost can be reduced, more specific suggestions on the application of system for large areas will be helpful.
Response:
• Lines 334-336 have been added to the paper to describe the deployment of the system and

sensors to a larger area.

• Approximate costs of an individual phenotyping cluster (with 10 distributed nodes and one server node) has been included in lines 191-197.

• The effective range of a star network and infrastructure requirements in terms of data storage have been added in lines 191-197 and line 234.

Close
