# Supplementary material for: CropSight: a scalable and open-source information management system for distributed plant phenotyping and IoT-based crop management
Source: Gigascience. 2019 Jan 31;8(3):giz009. doi: 10.1093/gigascience/giz009 (PMC6423370; doi:10.1093/gigascience/giz009)
Supplement: Reviewer_1_Report_Original_Submission -- Andri Nugroho — 11/19/2018 Reviewed [file giz009_reviewer_1_report_original_submission.pdf]

Reviewer Report

Title: CropSight: a scalable and open-source information management system for distributed plant phenotyping and IoT-based crop management

Version: Original Submission **Date:** 11/19/2018

Reviewer name: Andri Nugroho

Reviewer Comments to Author:

Title: CropMonitor: a scalable open-source experiment management system for distributed plant phenotyping and IoT-based crop management
1) Are the methods appropriate to the aims of the study, are they well described, and are necessary controls included?
The method for this study is well described, because this manuscript is about technical notes, so each stage of development should be mentioned clearly. However, in to improve this manuscript, herewith my recommendation:
a. The schematic system on Fig. 1, would be better if the flow of data and information provided in different arrow and colour, and the network typology should be briefly visualized.
b. The flowchart (Fig.2.C) of the data transmission from each node and server would be easier to understand if it can be visualized in completed flowchart, kindly refer the example on this paper (https://doi.org/10.1016/j.compag.2016.04.025)
c. Dealing with the utilization of camera in outdoor, is there any calibration method for white balance? Because the sunlight intensity is different every sampling. If there are any method to white balance adjustment it would be more useful.
d. The environmental sensor position during environmental measurement also should be standardized, if it will be used for estimating the reference Evapotranspiration (ETo), it should follow the standard on FAO56 Penmann Monteith

Level of Interest

Please indicate how interesting you found the manuscript: Choose an item.

Quality of Written English

Please indicate the quality of language in the manuscript: Choose an item.

Declaration of Competing Interests

Please complete a declaration of competing interests, considering the following questions:

- Have you in the past five years received reimbursements, fees, funding, or salary from an organisation that may in any way gain or lose financially from the publication of this manuscript, either now or in the future?
- Do you hold any stocks or shares in an organisation that may in any way gain or lose financially from the publication of this manuscript, either now or in the future?
- Do you hold or are you currently applying for any patents relating to the content of the manuscript?
- Have you received reimbursements, fees, funding, or salary from an organization that holds or has applied for patents relating to the content of the manuscript?
- Do you have any other financial competing interests?
- Do you have any non-financial competing interests in relation to this paper?

If you can answer no to all of the above, write 'I declare that I have no competing interests' below. If your reply is yes to any, please give details below.

I declare that I have no competing interest's below.

I agree to the open peer review policy of the journal. I understand that my name will be included on my report to the authors and, if the manuscript is accepted for publication, my named report including any attachments I upload will be posted on the website along with the authors' responses. I agree for my report to be made available under an Open Access Creative Commons CC-BY license (http://creativecommons.org/licenses/by/4.0/). I understand that any comments which I do not wish to be included in my named report can be included as confidential comments to the editors, which will not be published.

I agree to the open peer review policy of the journal

To further support our reviewers, we have joined with Publons, where you can gain additional credit to further highlight your hard work (see: https://publons.com/journal/530/gigascience). On publication of this paper, your review will be automatically added to Publons, you can then choose whether or not to claim your Publons credit. I understand this statement.

Yes Choose an item.
