# Supplementary material for: CropSight: a scalable and open-source information management system for distributed plant phenotyping and IoT-based crop management
Source: Gigascience. 2019 Jan 31;8(3):giz009. doi: 10.1093/gigascience/giz009 (PMC6423370; doi:10.1093/gigascience/giz009)
Supplement: Reviewer_2_Report_Original_Submission -- Bing Lu — 11/20/2018 Reviewed [file giz009_reviewer_2_report_original_submission.pdf]

**Reviewer Report**

**Title: CropSight: a scalable and open-source information management system for distributed plant phenotyping and IoT-based crop management**

**Version: Original Submission** **Date:** 11/20/2018

**Reviewer name: Bing Lu**

**Reviewer Comments to Author:**

This manuscript introduces a crop monitoring system that assessing almost real-time crop physiological features using multi-sensor data and providing support to farmers for precision farming. The authors reviewed existing crop monitoring systems and discussed their advantages and disadvantages, which can help readers to understand current technologies in this field. The manuscript also discussed the integration of multi-sensor data and analysis of a 'big data'. These are popular topics in the precision farming and this research provided insights on that. Overall, this is an interesting project and will contribute to the IoT-based farming and support various agricultural activities. However, there are a few concerns that need to be addressed before publication.Please see below specific comments.

1. Line 60-93, the introduction of different platforms is good. One concern is that the remote sensing imagery has long been recognized as an essential data source for evaluating crop properties over large areas (as sensors cannot be deployed to cover large areas), how the platforms mentioned here deal with remote sensing imagery and extract crop information?
2. Line 235, the authors described uploading images of crops to server and users can check the images to understand crop condition. Since there may be a large number of photos taken every day/week, manual evaluation would be labour intensive. Is that possible to add some software that can automatically analyze these images and provide results to the users?
3. The authors introduced extensively the integration or connection of various sensors in the system, but didn't describe clearly which specific sensors can be integrated (e.g., soil moisture sensor? Fertilizer sensor?), how to setup these sensors in the field, and how the data from sensors are analyzed. These information will help readers to further understand the operation of the monitoring system.
4. In the Discussion and Outlook section, specifically 343-357, the authors discussed the potential of applying the monitoring system in real world to solve various challenges, which is good. However, the authors didn't describe clearly the challenges in deploying the system for large areas. How many sensors and how much cost needed? Although the authors indicated in 370-391 that the system is scalable and the cost can be reduced, more specific suggestions on the application of system for large areas will be helpful.

**Level of Interest**

Please indicate how interesting you found the manuscript: Choose an item.

**Quality of Written English**

Please indicate the quality of language in the manuscript: Choose an item.

**Declaration of Competing Interests**

Please complete a declaration of competing interests, considering the following questions:

- Have you in the past five years received reimbursements, fees, funding, or salary from an organisation that may in any way gain or lose financially from the publication of this manuscript, either now or in the future?
- Do you hold any stocks or shares in an organisation that may in any way gain or lose financially from the publication of this manuscript, either now or in the future?
- Do you hold or are you currently applying for any patents relating to the content of the manuscript?
- Have you received reimbursements, fees, funding, or salary from an organization that holds or has applied for patents relating to the content of the manuscript?
- Do you have any other financial competing interests?
- Do you have any non-financial competing interests in relation to this paper?

If you can answer no to all of the above, write 'I declare that I have no competing interests' below. If your reply is yes to any, please give details below.

I declare that I have no competing interests

I agree to the open peer review policy of the journal. I understand that my name will be included on my report to the authors and, if the manuscript is accepted for publication, my named report including any attachments I upload will be posted on the website along with the authors' responses. I agree for my report to be made available under an Open Access Creative Commons CC-BY license (http://creativecommons.org/licenses/by/4.0/). I understand that any comments which I do not wish to be included in my named report can be included as confidential comments to the editors, which will not be published.

I agree to the open peer review policy of the journal

To further support our reviewers, we have joined with Publons, where you can gain additional credit to further highlight your hard work (see: https://publons.com/journal/530/gigascience). On publication of this paper, your review will be automatically added to Publons, you can then choose whether or not to claim your Publons credit. I understand this statement.

Yes Choose an item.
